# Pfeiffer syndrome type 3 with FGR2 c.1052C>G (p.Ser351Cys) variant in West Africa: a case report

**DOI:** 10.11604/pamj.2021.40.136.31395

**Published:** 2021-11-04

**Authors:** Kwadwo Apeadu Danso, Rosemary Sefakor Akuaku, Florence Naa Adoley Young, Samuel Agyei Wiafe

**Affiliations:** 1 Department of Paediatrics, Cape Coast Teaching Hospital, Cape Coast, Ghana,; 2 Rare Disease Ghana Initiative, Accra, Ghana

**Keywords:** Pfeiffer syndrome, proptosis, broad big toes, macrocephaly, case report

## Abstract

Pfeiffer syndrome is a rare genetic condition that includes anomalies of the head, hands, and feet. It was originally described by Rudolf Pfeiffer in 1964. As a result of varied clinical presentations, there is a low threshold for missing the diagnosis. Three (3) cases were found by the authors in the medical literature from the African continent, all of which lacked molecular studies. The main dysmorphic features we observed in our patient were; macrocephaly with widely gaped sagittal sutures, proptosis with ocular hypertelorism, ankylosed elbows, wide sandal gap and medially deviated broad great toes. In this case, sequence analysis using Illumina technology and deletion/duplication testing of 65 genes for variants associated with craniosynostosis syndromes was performed at Invitae Medical Genetic laboratory. A diagnosis of Pfeiffer syndrome type 3 with FGFR2 c.1052C>G (p.Ser351Cys) variant was made. In conclusion, this case will aid health care providers especially in areas of low accessibility to molecular studies to promptly identify, appropriately manage the condition as well as counselling the parents to offset the risk of abandonment of neonates with dysmorphic features.

## Introduction

Pfeiffer syndrome is a rare autosomal dominantly inherited disorder that is associated with craniosynostosis, broad thumbs and big toes, and partial syndactyly on hands and feet. It affects about 1 in 100,000 individuals [1]. Hydrocephaly may be found occasionally, along with severe ocular proptosis, ankylosed elbows, abnormal viscera, and slow neuro-cognitive development. Based on the severity of the phenotype, Pfeiffer syndrome has been divided into three clinical subtypes namely type 1, 2, and 3. Type 1 is referred to as the classic Pfeiffer syndrome and it presents with mild manifestations including brachycephaly, mid-face hypoplasia, and fingers and toes abnormalities. It is associated with normal neurological and intellectual development, and generally has a good outcome. Type 2 consists of tri-lobated skull deformity (cloverleaf skull), extreme proptosis, fingers and toes abnormalities, elbow ankylosis or synostosis, developmental delay, and neurological complications. Individuals with Pfeiffer syndrome type 3 have symptoms and findings similar to those present in Pfeiffer syndrome type 2, except for cloverleaf skull deformity. The major diagnostic clues in Type 3 remain classic Pfeiffer hands and feet in association with craniosynostosis, regardless of craniofacial variability or the presence or absence of visceral anomalies [2]. An overlap between the three types may occur. Mutations in the fibroblast growth factor receptor (FGFR) genes 1 and 2 have been implicated [3]. This will probably be the first reported case of Pfeiffer syndrome type 3 with an established molecular diagnosis from the West African sub-region and Africa at large.

## Patient and observation

**Patient information**: a female term neonate was delivered on February 20, 2021, at Cape Coast Teaching Hospital Ghana via emergency caesarean section on account of poor progress of labour with acute fetal distress. The birth weight was 3.8kg and Apgar scores were 2/10 and 5/10 at the first and fifth minute respectively. The baby was the 5^th^ child of a couple, a 39-year-old man and a 34-year-old woman, in a non-consanguineous marriage and with no known family history of congenital anomalies. From a family picture, no other nuclear family member was found to have similar physical features. The neonate was discharged after spending a total of 20 days on admission at the Special Baby Care Unit of the Hospital. The baby died 5 months later.

**Figure 1:**
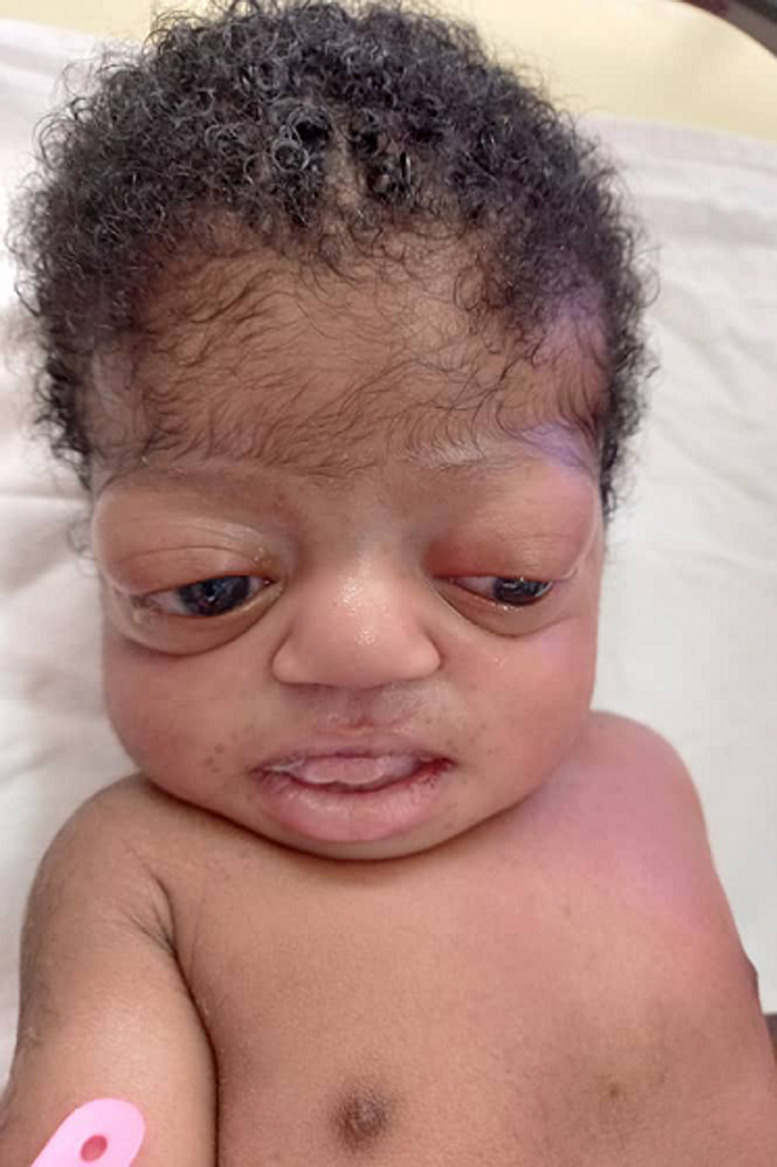
proptosis with ocular hypertelorism

**Figure 2:**
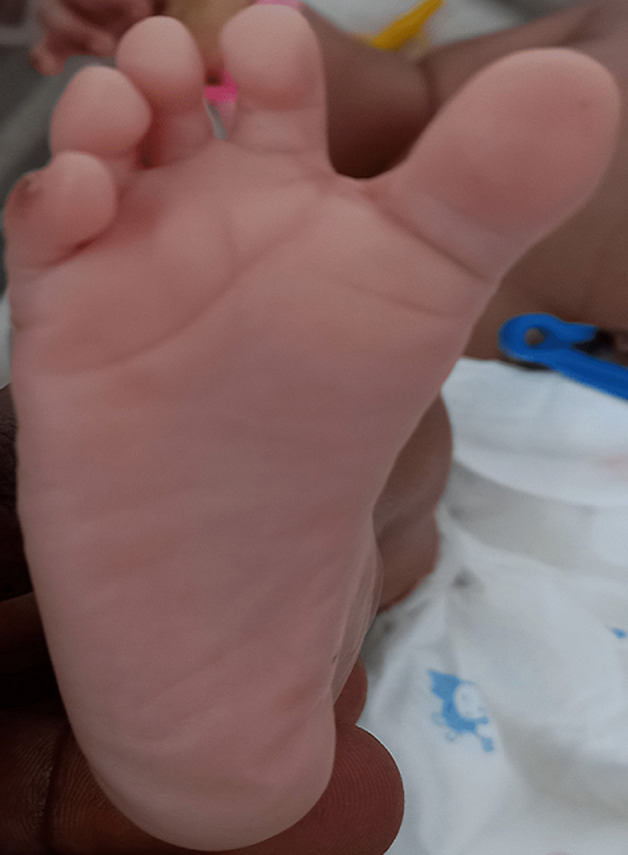
medially deviated broad right big toe with wide sandal gap

**Figure 3:**
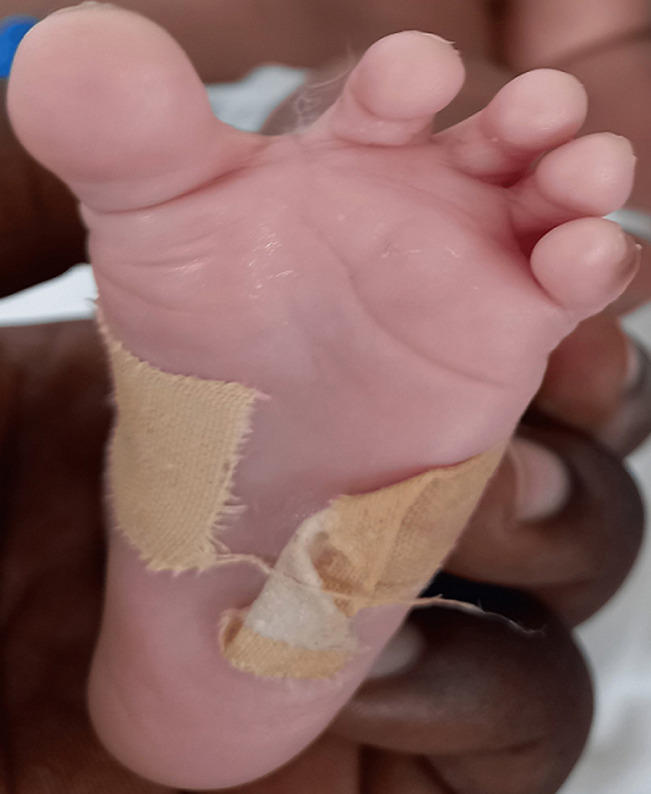
medially deviated broad left big toe with wide sandal gap

**Clinical findings**: the baby was noticed on the day of admission to the unit to have the following dysmorphic features; macrocephaly evidenced by an occipitofrontal circumference of 38.8cm (95^th^ centile) with widely gaped sagittal sutures, proptosis with ocular hypertelorism (Figure 1), low set ears, protruding tongue, mandibular prognathism, ankylosed elbows, sacral dimple, wide sandal gap and medially deviated broad great toes (Figure 2 and Figure 3). The clinical problems identified on initial assessment were respiratory distress which was evidenced by deep laboured breathing with poor oxygen saturation of 55% on room air, seizures of the upper limbs, high-grade fever, and pallor. Other examination findings identified were bilaterally fused coronal sutures. The parents were counselled on the child´s condition. The child was later noticed to be drooling most of the time and on that note, ENT was consulted.

**Diagnostic assessment**: an initial complete blood count showed an elevated WBC of 24.8 x 109/L with an accompanying increase in lymphocytes of 15.7 x 109/L; low hemoglobin 10.6g/dl, other parameters were normal. A repeat complete blood count done 6 days later showed an elevated WBC of 21.51 x 109/L with an accompanying increase in neutrophils of 12.48 x 109/L and low hemoglobin of 9.4g/dl with other parameters being normal. The results from both blood and urine culture and sensitivity tests yielded no bacterial growth. CSF bacteriology and biochemistry did not yield any significant findings. Liver function tests and serum electrolytes including calcium, magnesium and phosphate were essentially normal. Renal function was deranged with high serum creatinine and urea of 500.3µmol/L and 34.36mol/L respectively but normalized throughout the admission. Imaging investigations done included chest x-ray, bedside echocardiogram, and abdominal ultrasound, all of which yielded normal anatomical findings. A challenge encountered was the malfunctioning of the CT scan machine at the hospital.

**Diagnosis**: Pfeiffer syndrome among other diagnoses such as Apert and Crouzon syndromes was initially considered. Other diagnoses were early-onset neonatal sepsis complicated by severe anaemia, moderate hypoxic-ischaemic encephalopathy, acute kidney injury and upper airway obstruction. The final diagnosis was Pfeiffer syndrome type 3 with FGR2 c.1052C>G (p.Ser351Cys) variant.

**Therapeutic interventions**: IV phenobarbitone and phenytoin were administered to control the seizures; IV benzylpenicillin 0.38mu twice daily and gentamicin19mg 36 hourly were administered for the first 5 days, then switched to IV flucloxacillin 190mg twice daily and cefotaxime 190mg twice daily for the subsequent 7 days due to unsettling fever. Finally, IV meropenem 68mg for 6 days was given. Oxygen support was administered via CPAP and the baby was haemotransfused.

**Follow-up and outcome of interventions**: fever finally settled after completing 6 days of intravenous meropenem, she was weaned off supplemental oxygen support as respiratory distress improved, and feeding was established orally via a nasogastric tube gradually increased to even surpass the daily fluid requirement. Baby graduated from feeding via nasogastric tube to cup feeding. She was discharged with scheduled follow-up visits. The baby was seen twice on an outpatient basis and her condition was satisfactory. A blood sample sent for molecular studies at Invitae Medical Genetic laboratory revealed a heterozygous pathogenic variant in intron 8 of the FGFR2 gene, c.1052C>G (p.Ser351Cys). This result came in after we had received a phone call from the parents on August 9, 2021, about the death of the child the day before. The child was reported to have become lethargic and died some hours after reporting to the district hospital. An autopsy was not done. The parents have been counselled on the findings of the genetic study.

**Patient perspective**: the parents were counselled on a tracheostomy for the baby. However, they changed their mind a day afterwards because they were doubtful of the successful outcome of the procedure. On day 12 of admission, the parents opted for the baby to be discharged against medical advice on account of poor progress in the condition. The mother of the child even threatened to abandon her if their wish was not granted. The couple was taken through another extensive education and counselling and assured of discharge once the baby's clinical condition improved significantly.

**Informed consent**: an informed consent for photographic documentation and anonymous use as well as genetic testing was sought and obtained from the mother of the baby. The consent forms used were obtained through the Rare Disease Ghana Initiative diagnostic program.

## Discussion

The diagnosis of Pfeiffer syndrome is based on the presence of craniosynostosis and abnormal thumbs and/or big toes. Due to the wide clinical variability, a molecular study is seen as an important complement to the clinical phenotype to confirm the diagnosis [1]. The clinical features we are reporting is in accordance with Pfeiffer syndrome type 3 because although the thumbs looked normal, the baby had medially deviated broad great toes which is a major diagnostic clue [1]. Cases of PS type 3 with normal thumbs have been reported by Kerr *et al*. [4] and Gripp *et al*. [5]. Other significant features of our patient were proptosis, hypertelorism, elbow ankylosis and macrocephaly which was not tri-lobulated. In West Africa, Badoe [6] has reported two suspected cases of Pfeiffer syndrome subtype 3 over a 10-year period. The common dysmorphic features he identified in both cases were; proptosis, hypertelorism, flat nasal bridge, broad thumbs and medially deviated broad big toes. A significant negative finding in both cases was the absence of a cloverleaf skull. These features except for the flat nasal bridge and broad thumbs were found in our case. From East Africa, Amiji *et al*. [7] in 2020 reported on a case of Pfeiffer syndrome type 2 where a cloverleaf skull was identified, a differentiating feature between type 2 and 3 [2].

The mutation identified in our patient, Ser351Cys in FGFR2 replaces serine with cysteine at codon 351 of the FGFR2 protein. This mutation was found in the first reported case of Pfeiffer syndrome type III [5]. Some of the clinical features shared by that patient and ours were; hydrocephalus, extreme proptosis, bilateral elbow ankylosis, deviated first toe and normal looking thumbs. Some studies have reported that these mutations originate from the chromosome 8p11.22-P12 in the FGFR1 gene and the chromosome 10q25-q26 in the FGFR2 gene [8]. Identical mutations in the fibroblast growth factor receptor gene have been reported in other craniosynostosis deformities such as Crouzon and Jackson-Weiss syndrome resulting in variable expression with distinct phenotypes. Nevertheless, they do not have hand and foot abnormalities as in Pfeiffer syndrome. Pfeiffer and Apert syndromes are noteworthy for some similarities, however, the presence of proptosis, in this case, speaks for the former. In terms of limitations, a head CT could not be done.

The FGFR1 and FGFR2 genes play an important role in signalling the cell to respond to its environment, perhaps by dividing or maturing. A mutation in either gene causes prolonged signalling, which can promote the early maturation of bone cells in a developing embryo and the premature fusion of bones in the skull, hands, and feet. The syndrome is genetically heterogeneous, the aetiology is autosomal dominant or fresh mutations in type I conditions and sporadic in types 2 and 3. Due to the absence of similar physical features in the rest of the family, a de novo mutation could be a likely explanation. The father was 39 years old at the time the patient was born. Glaser *et al*. [9] have shown that there is an advanced parental age effect of paternal origin for sporadic cases of Pfeiffer and Crouzon syndromes. In their study, advanced paternal age was noted for the fathers of patients with Crouzon syndrome or Pfeiffer syndrome as compared to the fathers of control individuals (34.50±7.65 years vs. 30.45±1.28 years, P<.01). To date, all cases of Type 2 and 3 have occurred sporadically, their overall prognosis being very poor with early death. Similarly, our patient was 5 months of age when she died. In terms of limitation, a head CT scan could not be done in our case due to a malfunction of the equipment at the hospital.

## Conclusion

Combining the clinical features and molecular studies makes Pfeiffer syndrome type 3 the most likely diagnosis amongst all the other craniosynostosis syndromes. In terms of clinical practice, this case will aid health care providers especially in areas of low accessibility to molecular studies to promptly identify, appropriately manage the condition as well as counselling the parents to offset the risk of abandonment of neonates with dysmorphic features.
